# Veno-venous extracorporeal membrane oxygenation in the management of refractory bilateral bronchial dehiscence after lung transplant: a case report

**DOI:** 10.1186/s40981-021-00480-6

**Published:** 2021-10-16

**Authors:** Makiko Tani

**Affiliations:** Department of Anesthesiology and Resuscitology, Graduate School of Medicine Dentistry and Pharmaceutical Sciences, Okayama University, 2-5-1, Shikata-cho, Kita-ku, Okayama, 700-8558 Japan

**Keywords:** Bronchial dehiscence, Extracorporeal membrane oxygenation, Lung transplant

## Abstract

**Background:**

Bronchial dehiscence is a life-threatening complication after lung transplant. If it is not treated by placement of stent or reanastomosis, the chance of survival will depend on the availability of a new graft. However, retransplant is not a practical management option in Japan, where waiting time for lung transplant is extensive. We described a case of refractory bilateral bronchial dehiscence managed by veno-venous extracorporeal oxygenation membrane (VV ECMO) while allowing the dehiscence to heal.

**Case presentation:**

A 25-year-old man with idiopathic pulmonary arterial hypertension underwent a bilateral lung transplant. The patient developed bilateral bronchial dehiscence. Open reanastomosis was not successful, and air leakage recurred under low positive pressure ventilation. VV ECMO was established to maintain oxygenation with spontaneous breathing until both dehiscence were closed by adhesions.

**Conclusion:**

In a patient with refractory bilateral bronchial dehiscence, VV ECMO may provide bronchial rest and serve as a bridge therapy to recovery.

## Background

Bronchial dehiscence is one possible anastomotic airway complication after lung transplant. Bronchial dehiscence has been reported in 1–10% of lung transplant recipients [1], but it is associated with high mortality [2]. Treatment options for bronchial dehiscence are placement of stents, surgical suture, pneumonectomy, and retransplant [3]. Indication of the options should be considered based on estimated effectiveness, operability, and availability for new lung graft.

Pulmonary infections after lung transplant predispose patients to bronchial dehiscence [3]. Impaired tissue oxygenation and carbon dioxide elimination because of pneumonia necessitate mechanical ventilation, which increases the risk of bronchial dehiscence.

Here, we report a case of bilateral bronchial dehiscence with pneumonia, in which none of the management options for bronchial dehiscence described above was effective or applicable. We utilized veno-venous extracorporeal membrane oxygenation (VV ECMO) until the dehiscence closed in order to prevent air leak and maintain oxygenation and ventilation.

## Case presentation

We obtained a written informed consent from the patient for the publication of this case report.

**Fig. 1:**
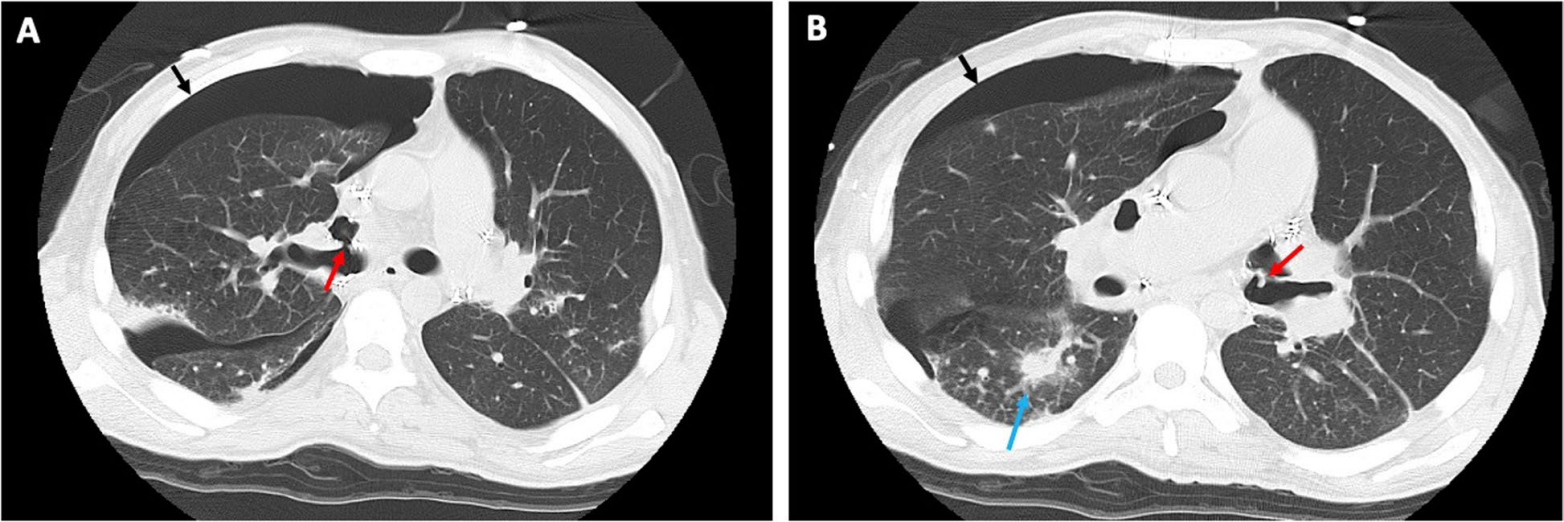
**A**, **B** Images of computed tomography scan performed 15 days after the transplantation showing bronchial dehiscence (red arrows), pneumothorax (black arrows), and associated pneumonia (blue arrow)

A 25-year-old male with idiopathic pulmonary arterial hypertension underwent double lung transplant. He was extubated on postoperative day (POD) 4 uneventfully other than 2 days being febrile. However, CT scan on POD 6 revealed filtration in both lungs. We diagnosed the filtration as pneumonia due to Pseudomonas aerginosa, Enterobacter, and methicillin-resistant Staphylococcus aureus, and administered antibiotics specific to the bacteria. On POD 17, a chest X-ray revealed right-sided pneumothorax, and a chest tube was inserted into the right chest cavity. A subsequent CT scan revealed bilateral bronchial dehiscence (Fig. 1) and adhesion of pulmonary arteries (PAs) into the bronchus.

The patient was transferred to the operating room for emergency surgical inspection. With ECMO on standby, general anesthesia was induced. The trachea was intubated with a double lumen tube. Bronchoscopy was performed before surgery which revealed dehiscence of bilateral bronchial anastomosis.

Surgical inspection revealed both-sided bronchial dehiscence and adhesion of the left PA to the left bronchus. Due to a risk of PA penetration into the bronchus, the dehiscence could not be sutured circumferentially. Then, the dehiscence was repaired with partial suture combined with omentoplasty and thymus wrapping under mechanical ventilation. We did not establish ECMO or cardiopulmonary bypass due to concerns that bleeding from the PA could become uncontrollable due to anticoagulation. A tracheostomy was also performed at the end of the surgery. There was no pneumothorax or air leakage from the bilateral chest tubes.

After the surgery, we attempted to wean the patient from ventilator support to minimize positive pressure on the repair site. However, respiratory suppression by high dose sedatives (propofol 250 mg/h, morphine 6 mg/h, and dexmedetomidine 0.5 mcg/kg/h) to prevent coughing and impaired oxygenation due to pneumonia necessitated positive airway ventilation (positive end-expiratory pressure 3–5 cmH_2_O, pressure control 5–8 cmH_2_O) with inhaled nitric oxide. The patient spent 3 days with no air leakage. On POD 21, massive air leakage appeared from both chest tubes under peak inspiratory pressure (PIP) 13 cmH_2_O. Ineffective ventilation due to air leakage as well as pneumonia deteriorated the gas exchange to PaO_2_ 70 mmHg, PaCO_2_ 100 mmHg (F_I_O_2_ 1.0, positive end-expiratory pressure 8 cmH_2_O, PIP 15 cmH_2_O). Hypercarbia necessitated emergent installation of VV ECMO through both femoral veins, and positive airway ventilation was discontinued. The patient was assisted with VV ECMO while breathing spontaneously through the tracheostomy, resulting in improved gas exchange and cessation of air leakage.

Placement of stents and re-opening for exploration were management options for the dehiscence; however, we decided neither was indicated for the patient because of PA adhesion and concern of subsequent PA perforation into the bronchus. Retransplant for this patient was theoretically a treatment option; however, retransplant was not practical because of the paucity of lung donors in Japan.

During ECMO support, we tolerated partial flow (60–70% of total flow and 4L/min sweep gas) as to not administer excess volume and to keep the lungs dry. We also used high flow oxygen therapy (F_I_O_2_ 1.0 and oxygen flow rate 20 L/minute) for spontaneous breathing support and preventing lung collapse, and PaO_2_ 80s mmHg and PaCO_2_ 40s mmHg were maintained. In addition, activated clotting time (ACT) was controlled at approximately 150 seconds to prevent bleeding particularly from the anastomosis site and tracheostomy.

Under VV ECMO support, we provided conservative therapy for the patient. Intravenous morphine was continued for suppressing cough and avoiding high pressure on the repair lesions. Instead, sputum was removed with bronchoscopy every four hours. We awakened the patient in the daytime and encouraged physical therapy to prevent muscle atrophy and improve pulmonary function. Antibiotics were continued for pneumonia.

**Fig. 2:**
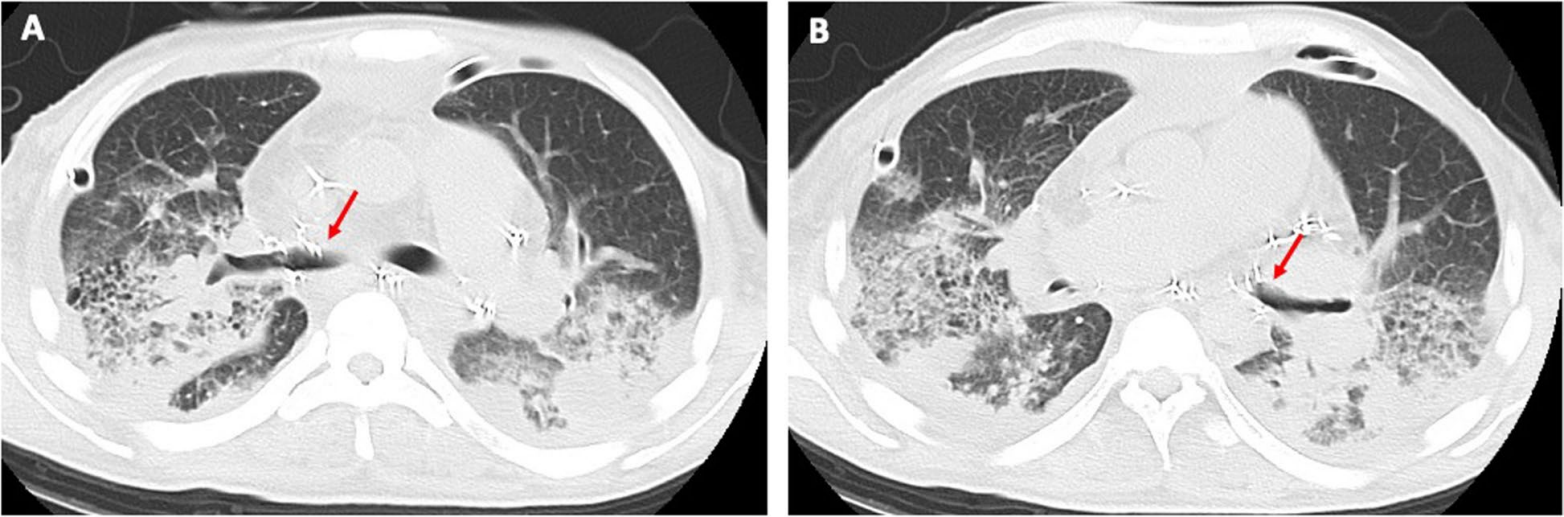
**A**, **B** The follow-up CT scan 12 days after the surgical repair revealing good healing of the bilateral dehiscence (red arrows)

On POD 29, improvement of bronchial dehiscence was confirmed by bronchoscopy and CT scan (Fig. 2A, B); however, bilateral pneumonia remained. After the pneumonia improved, the patient was successfully weaned from ECMO on POD 32, the twelfth day after ECMO installation. The patient could maintain oxygenation (PaO_2_ 81mmHg) and carbon dioxide removal (PaCO_2_ 38mmHg) by high-flow oxygen therapy (F_I_O_2_ 0.9 and oxygen flow 20L/minute). ECMO circuit exchange was necessary twice due to blood clots within the artificial lung during the 12 days. However, the patient had neither embolic nor hemorrhagic complications. He was discharged from the intensive care unit after 1 month.

## Discussion

This is a case of bilateral bronchial dehiscence with respiratory failure which resolved after 12 days of VV ECMO support, despite the fact that the sites of dehiscence were not completely repaired surgically.

Management of patients with repaired bronchial dehiscence and respiratory failure is challenging because there is a management dilemma. Airway pressure should be minimized to protect repaired anastomosis, whereas adequate respiratory support is required to maintain oxygenation and ventilation. Implementing VV ECMO for patients with bronchial dehiscence has two benefits: (1) Influence on the surgical repair of the bronchial dehiscence by inspiratory positive airway pressure can be reduced. Positive airway pressure decreases pulmonary blood flow and worsens bronchial ischemia [4]. In addition, reduction of pulmonary shear stress has a protective effect on bronchial healing [5]. (2) Reduced oxygenation and carbon dioxide elimination induced by respiratory failure were substituted.

There is no clear consensus on treatment for bronchial dehiscence, and management practice depends on the severity of the dehiscence and associated complications [6]. For cases with large dehiscence or cases of failed stent placement, surgical repair is considered. However, surgical repair in such cases is difficult due to the presence of infection and ischemia, resulting in high failure rate and poor outcome [6, 7]. If the management mentioned above is not successful, allograft resection or re-transplant could be therapeutic options. In this case, these strategies were considered when air leakage recurred after surgical repair; however, neither strategy was adopted. The bronchial dehiscence was both-sided, and both grafts were affected by an infection. A one-sided allograft resection would not have been tolerable. Furthermore, the waiting time for a lung transplant did not depend on the allocation score in Japan, as the average waiting time was about 900 days in Japan. Thus, emergency re-transplant was not a viable option.

For reviving the impaired allografts, management strategy during ECMO support was critical. First, we set the lower ACT goal of heparinization for ECMO than the popular ACT range of 180-200 seconds with the aim of preventing hemorrhage [8]. Hemorrhage from anastomosis and tracheostomy could be life-threatening in this case. Second, we started physical therapy while the patient was on ECMO support to improve respiratory function. There are some complications from physical therapy combined with ECMO, such as a decrease in peripheral oxygen saturation, cannula fracture, and obstructive thrombus in return cannula [9]; however, physical therapy together with ECMO is relatively safe and effective for secretion clearance and pulmonary recovery [10, 11].

We did not implement VV ECMO during the surgical repair considering the risk of bleeding from the PA which was firmly adhered to the bronchus. We should have used VV ECMO right after the surgical repair was completed, an action that could have prevented recurrent air leakage which was induced by inspiratory airway pressure affecting the reanastomosis.

## Conclusion

Bronchial dehiscence after lung transplant is life-threatening. In our case, VV ECMO provided bronchial rest and served as a bridge therapy to recovery.

## Data Availability

Not applicable due to patient privacy concerns.

## Abbreviations

VV ECMO: Veno-venous extracorporeal oxygenation membrane
POD: Postoperative day
PA: Pulmonary artery
PIP: Peak inspiratory pressure
PaO_2_: Arterial partial pressure of oxygen
PaCO_2_: Arterial partial pressure of carbon dioxide
F_I_O_2_: Fraction of inspiratory oxygen
ACT: Activated clotting time
